# Management practices related to the control of gastrointestinal parasites on Swedish pig farms

**DOI:** 10.1186/s40813-021-00193-3

**Published:** 2021-01-20

**Authors:** Emelie Pettersson, Marie Sjölund, Torun Wallgren, Eva Osterman Lind, Johan Höglund, Per Wallgren

**Affiliations:** 1 National Veterinary Institute, SVA, 751 89 Uppsala, Sweden; 2 Department of Clinical Sciences, Swedish University of Agricultural Sciences, Box 7054, 750 07 Uppsala, Sweden; 3 Department of Animal Environment and Health, Swedish University of Agricultural Sciences, Box 7076, 750 07 Uppsala, Sweden; 4 Department of Biomedical Science and Veterinary Public Health, Swedish University of Agricultural Sciences, Box 7036, 750 07 Uppsala, Sweden

**Keywords:** Antiparasitic drugs, Anthelmintic, Biosecurity, Hygiene, Parasite control, Questionnaire, Survey

## Abstract

**Background:**

Internal parasites are common in pigs worldwide and may induce clinical disease or subclinical infections with negative effects such as poor weight gain and reduced welfare, which in turn affect productivity. Effective parasite control to reduce the negative impact of parasitic infections demands a combination of antiparasitic drugs as well as various hygiene and biosecurity practices. The aim of this study was to obtain information on current management practices and parasite control routines used on Swedish pig farms using an online questionnaire.

**Results:**

Antiparasitic drugs were used on 69% of the farms routinely and were mainly administered to sows just prior to farrowing. Less than 5% of the herds conducted faecal analysis for parasites. Batchwise, age segregated rearing was common and overall, it was practiced for piglets, growers, and fatteners on 88, 80 and 75% of the farms, respectively. Large and medium sized farms appeared to apply stricter hygiene and biosecurity measures to the growing pigs compared to small farms. Dry sows were mainly housed in groups on deep litter straw beds and cleaning, as well as disinfection, between each group was less common compared to what was practiced for growing pigs. Outdoor access was rare and only occurred on organic and small farms. Most of the farms, 54, 74 and 82% of small, medium, and large sized herds respectively, reported to have less than 5% white spot lesions, caused by migrating *A. suum* larvae, registered at slaughter.

**Conclusion:**

Several risk factors for parasite infections, such as bedding material, group housing and solid floors, are mandatory requirements by national law. However, it was evident from this study that although strategic hygiene and biosecurity practices appeared common, they were not practiced in all herds and less so for dry sows. Antiparasitic drugs were used frequently and mainly through routine prophylactic treatments without prior testing for parasites. A holistic approach is necessary when designing efficient parasite control programs, and it is essential that management factors and routine monitoring of parasites are given attention. This to achieve efficient parasite control and reduce the risk of unnecessary use of antiparasitic drugs.

**Supplementary Information:**

The online version contains supplementary material available at 10.1186/s40813-021-00193-3.

## Background

In Sweden there are approximately 1300 registered pig producers and out of these 61% keep sows for breeding and 79% produce fatteners. Approximately 58% of the registered sow holdings had a herd of less than 100 sows, 32% had 100–500 sows and 10% had more than 500 sows. Around 2.6 million pigs are slaughtered annually [1]. The vast majority of all pig farms in Sweden are conventional and located in the southern and central parts of the country. According to Swedish legislation, pigs are to always be loose-housed, fully slatted floors are not allowed, and manipulative rooting material must be provided in all production systems. Weaning is generally not done before 28 days of age [2]. Use of low-dosed antibiotics for growth promotion has been banned since 1986 [3]. Approximately 2 % are registered as Specific Pathogen Free (SPF) farms and 2 % are organic [4]. On organic pig farms, year round outdoor access must be provided [5]. Batchwise, or age segregated, rearing of growing pigs is achieved when groups of sows are simultaneously weaned and inseminated after 4 to 7 days post weaning when coming into heat. Following group housing in the dry sow section, sows are transferred to an empty farrowing unit a few days prior to farrowing. Sows remain there until weaning when they return to the mating unit. All piglets in a batch are reared to market weight without mixing with pigs of any other age group [6]. In contrast, in continuous production systems different age categories of growing pigs are mixed.

Gastrointestinal parasites are common in pigs worldwide, and their effects depend on the parasite burden and host responses [7]. Previous studies have shown that the most common parasites in modern pig production are the intestinal nematodes *Ascaris suum*, *Oesophagostomum* spp. and *Trichuris suis*, as well as the coccidia *Cystoisospora suis* and *Eimeria* spp. [8]. The prevalence is generally dependent on host age where *C. suis* is commonly found in piglets, *A. suum* and *T. suis* in growing animals and *Oesophagostomum* spp. and *Eimeria* spp. in adults [8–10]. Transmission of all these parasites is faecal-oral and direct via eggs containing an infective third stage larva (L3) as for *A. suum* and *T. suis,* free living L3 as for *Oesophagostomum* spp., or sporulated coccidian oocysts shed in the environment [11–13]. The parasites can cause clinical gastrointestinal disease or more commonly subclinical infections, which in turn may lead to reduced weight gain, feed conversion and welfare [7, 14, 15]. Condemnation of internal organs at slaughter, especially livers with milk spots due to the migration of *A. suum* larvae, may contribute further to economic losses [16].

To reduce the negative effects of parasites in a pig herd, adequate parasite control methods are essential. However, using antiparasitic drugs alone has been concluded insufficient [11, 15, 17, 18]. Parasite control should be focused on both eliminating the parasites from the host as well as minimising the survival and transmission of parasites in the environment. Thus, effective parasite control requires an integrated approach combing the use of antiparasitic drugs and various management practices [9, 18]. There is however a growing concern for the development of resistance to antiparasitic drugs. In Sweden fenbendazole and ivermectin are the only drugs available for anthelmintic treatment of pigs, whereas toltrazuril is available for prevention of *C. suis* in piglets. Globally there are to date no reports of anthelmintic resistance in *A. suum* or *T. suis,* but there are several reports of drug resistance in *Oesophagostomum* spp. [19–23]. Resistance to toltrazuril has also been identified in *C. suis*, highlighting the necessity of responsible and more restrictive use of anticoccidial drugs [24, 25].

Although organic pig farms were investigated in 2008 [26], no systematic studies on parasite occurrence or parasite control measures in conventional pigs have been carried out in Sweden since the 1980’s [8]. Since then, conventional pig production systems have changed significantly and now include fewer but larger production sites [4]. These changes have also resulted in improved biosecurity measures [3].

To develop sustainable recommendations regarding parasite control, it is essential to document current management strategies that may have an impact on the parasitic burden in a herd. The aim of this study was therefore to obtain information on current management practices and parasite control routines used on Swedish pig farms.

## Methods

### Questionnaire

An online questionnaire was designed and distributed using the web-based service Questback Essentials (QuestBack Sweden Ltd., Stockholm, Sweden). Prior to release, the questionnaire was evaluated by veterinarians from the three national pig health organisations (Farm & Animal Health; Lunden Animal Health; and the District Veterinarians organized by the Swedish Board of Agriculture) for input. The questionnaire included 30 questions regarding farm structures, general husbandry practices, cleaning routines, deworming practices as well as health parameters. Twenty-nine questions were close-ended with specific response alternatives and one question was open-ended. Where appropriate, the questions included the option of replying “other” or “do not know”.

Information about the study and an invitation to participate were initially sent out via email through the three national pig health organisations (see above) to 816 registered pig farms. These were all the farms registered with the three pig health organisations and hence represented the majority of the commercial pig holdings in Sweden. No other selection or stratification into what farms were contacted was done. In addition, the project and a link to the online questionnaire were advertised in the national journal for pig producers, as well as in suitable groups on social media. Two reminders were sent out through the same channels. Farmers were anonymous in the questionnaire. The questionnaire stayed open between April 2018 and April 2019. Collected data were handled at the National Veterinary Institute (SVA) in accordance with the General Data Protection Regulation (GDPR).

### Data analysis

Raw data from the questionnaire was provided in an Excel file by Questback Essentials. All questionnaires were controlled manually and of those that had been submitted more than once from the same farm, only the first submission was included.

Data were analysed by examining descriptive statistics using Microsoft Excel and SAS® software version 9.4 (SAS Inst. Inc., Cary, NC, USA), as well as applying Chi-square tests when analysing categorical variables and the expected values were ≥ 5. Results were considered statistically significant if *p* < 0.05. When results were presented as percentages, they were calculated based on the number of farms where the question was applicable. For clarity, proportions are also shown when deemed necessary. It was possible to select multiple options for several questions, so the total response rate for some questions could be more than 100%.

When comparing management parameters in relation to herd size, results were often uniform and the criteria for statistical analysis using chi square tests were not fulfilled. When that was the case, only descriptive data is shown and in tables, this data is marked in italics.

## Results

### Farm characteristics

A total of 174 farms responded, giving a response rate of 21% (174/816). However, all 30 questions were not always answered. Of the responding farms, 78% (135/174) were sow holding farms, including 79 farrow-to-finish farms and 49 specialised piglet producers, selling growers at an approximate weight of 30 kg. The sow holding farms also included seven central units in sow pools (multi-site production), and five out of these seven central units also had farrowing facilities either selling the reared piglets as growers, at the approximate weight of 30 kg (*n* = 4) or rearing them to market weight (*n* = 1).

**Fig. 1:**
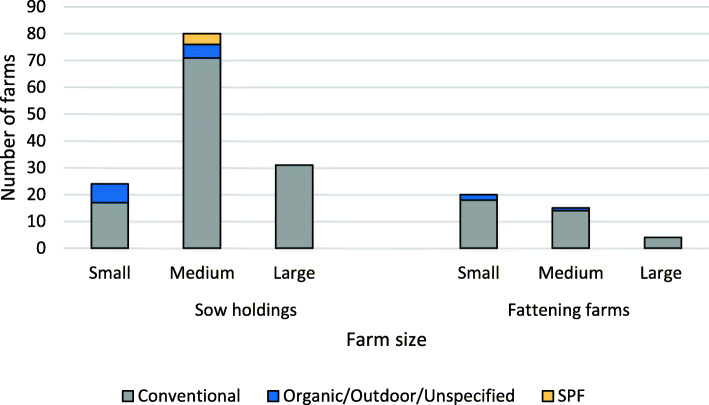
Type of production in relation to herd size as reported by the 174 surveyed Swedish pig farms. The figure shows 135 sow holdings, including central units in sow pools (small sized if fewer than 100 sows or less than 220 annual farrowings in a sow pool, medium sized if 100–400 sows or 220–880 annual farrowings in a sow pool and large sized if more than 400 sows or 880 annual farrowings in a sow pool) as well as 39 specialised fattening producers (small, medium sized and large when annually producing < 5000, 5000–10,000 and > 10,000 pigs, respectively). SPF: Specific pathogen free

The sow holding farms were divided into three categories: i) small herds (*n* = 24), ii) medium herds (*n* = 80) and iii) large herds (*n* = 31). The remaining farms (*n* = 39) were specialised fattening farms, purchasing growers at the approximate weight of 30 kg. Fourteen farms (8%) were organic and four (2%) were SPF farms (Fig. 1). Multisite production in terms of central units and/or satellites in sow pools, with either farrow-to-finish or piglet production, was carried out in 13% (23/174) of the responding herds.

### Husbandry practices

Overall, 88% (116/132) of farms practiced strict batch-wise production for piglets, 80% (106/132) for growers and 75% (89/119) for fatteners. Strict age-segregated batch-wise production was numerically more common in medium and large farms compared to small farms (Table 1).

**Table 1:** General husbandry practices reported by the surveyed Swedish pig farms, divided by age group and farm size. The farms were divided based on herd size where sow holdings were classified as small if fewer than 100 sows, medium sized if 100–400 sows and large if more than 400 sows. Specialised fattening herds were classified as small, medium sized and large when annually producing < 5000, 5000–10,000 and > 10,000 pigs, respectively. Regarding total numbers, different letters (a-c) within a column indicate a statistical difference (*p* < 0.05) between age categories. Regarding herd sizes, different letters (d-e) within a row indicate a statistical difference (*p* < 0.05) between herd sizes. The differences were compared statistically using Chi-square tests. When numbers are written in italics, the distribution of the responses did not allow for chi-square analysis

	Total		Herd size					
			Small		Medium		Large	
	n	%	n	%	n	%	n	%
**Strict batch wise production**								
Piglets	116/132	**88**^**b**^	*15/24*	***63***	*74/80*	***93***	*27/28*	***96***
Growers	106/132	**80**^**b**^	12/24	**50**^**d**^	70/80	**88**^**e**^	24/28	**86**^**e**^
Fatteners	89/119	**75**^**a**^	*22/33*	***67***	*57/74*	***77***	*10/12*	***83***
**Outdoor access**								
Piglets	13/132	**10**	*9/24*	***38***	*4/80*	***5***	*0/28*	***0***
Growers	12/132	**9**	*8/24*	***33***	*4/80*	***5***	*0/28*	***0***
Fatteners	10/119	**8**	*8/33*	***24***	*4/74*	***5***	*0/12*	***0***
Sows	14/135	**10**	*10/24*	***42***	*4/80*	***5***	*0/31*	***0***
**Liquid feed**								
Growers	78/132	**59**^**b**^	5/24	**21**^**d**^	54/80	**68**^**e**^	19/28	**68**^**e**^
Fatteners	97/119	**82**^**a**^	*15/33*	***45***	*70/74*	***95***	*12/12*	***100***
Sows	90/135	**67**^**b**^	4/24	**17**^**d**^	59/80	**74**^**e**^	27/31	**87**^**e**^
**Water source only over slatted floor**								
Farrow pens	68/132	**52**^**a**^	8/24	**33**^**d**^	47/80	**59**^**e**^	13/28	**46**
Grower pens	104/132	**79**^**b**^	10/24	**42**^**d**^	71/80	**89**^**e**^	23/28	**82**^**e**^
Fattener pens	93/119	**78**^**b**^	18/33	**55**^**d**^	65/74	**88**^**e**^	*10/12*	***83***
Dry sow pens	28/135	**21**^**c**^	*1/24*	***4***	*17/80*	***21***	*10/31*	***32***

Outdoor access was provided in all organic herds and in six non-organic herds. All non-organic herds that provided outdoor access were small-sized. Outdoor access appeared more common in small herds, but this could not be verified statistically due to the low number of herds with outdoor access in medium and large sized herds.

Data on the housing for the piglets were obtained from 132 farms. The most common type of farrowing pens were conventional pens with partly slatted floors (84%) where both the sow and piglets were moved at weaning. Thirteen farms (10%) reported to use family pens, where several sows were housed together with their piglets. One farm (< 1%) used a farrow-to-finish pen and the remaining farms used unspecified pens. Straw was used as bedding material in the farrowing pens on 92% of the farms, 55% used wood shavings and 21% used peat. Ten percent reported to use deep litter straw beds.

A weaning age of 4–5 weeks was reported on 67% of the farms, all which had conventional production. A weaning age of 5–6 weeks was reported on 24% of the farms and out of those (*n* = 32), one farm was organic, and 31 farms had conventional production. A weaning age of 6–7 weeks was practiced on 9% of the farms (*n* = 12), ten farms with organic production and two with conventional production. One outdoor herd weaned the piglets when older than 7 weeks. Piglets were moved from the farrowing pens directly at weaning in 43% of the small farms, 64% of the medium farms and in 81% of the large farms. At weaning, 39% of the farms reported to always apply high-dosed zinc oxide in the feed to prevent post weaning diarrhoea. Among these, 2% reported to use it sometimes and the remaining 59% had not done so in the past 12 months or never.

Data on the housing for the growers were obtained from 132 farms. Post weaning, conventional pens with partly slatted floors were used on 71% of the farms. Larger farrow-to-grower pens, where the growers remained with their litter mates in the farrowing pen from weaning, were used on 16% of the farms, whereas 12% kept the growers in large pens with several litters mixed and < 1% used farrow-to-finish pens. Straw was used as bedding material in the grower pens in 79%, wood shavings in 47%, peat in 18% of the farms. Growers stayed in the grower units up to 8 (6%), 9–10 (16%), 11 (33%), 12 (28%) or 13–14 (17%) weeks of age.

Data on the housing for the fatteners were obtained from 119 farms. Conventional pens with partly slatted floors were used on 83% of the farms; deep litter straw beds were used on 6% of the farms; < 1% used a farrow-to-finish pen and 10% used an unspecified pen type. Straw was used as bedding material in the fattening pens in 81%, wood shavings in 74% and peat in 7% of the herds. Four percent reported to use “other”.

Data on the housing for the dry sows were obtained from 135 farms. Dry sows were group-housed in continuous production systems from mating until transfer to the farrowing pens, either in i) deep litter straw pens (53%), ii) conventional pens with partly slatted floors and limited bedding material (9%), iii) a combination of both pen types (24%) or iv) unspecified pens (14%). In conventional pens, straw was used as bedding material in 60%, wood shavings in 15% peat in 3% and the remaining farms reported “other”.

Liquid feed was used alone or in combination with dry feed for fatteners on 82% of the farms, which was more common than for growers (59%) and dry sows (67%) (*p* < 0.05). As seen in Table 1, it also appeared to be more common to feed liquid feed on medium and large sized farms, but this could not be verified statistically.

Nipple drinkers were predominantly used to supply water and were used in 83% of the farrowing units, 87% of the grower units, 91% of the fattening units and for 49% of the dry sow units. The remaining farms used automatic waterers alone, or in combination with nipple drinkers. One fattening farm with outdoor production reported to use water troughs. The water source was more frequently placed over the slatted part of the floor in the grower (79%) and fattening units (78%) compared to in the farrowing units (52%) and the dry sow units (21%) (*p* < 0.05). Numerically, medium and large sized farms reported to more frequently place the water source over the slatted part of the floor compared to small farms (Table 1).

### Biosecurity practices

Cleaning of empty boxes between batches was more commonly (*p* < 0.05) practiced in the farrowing units (80%) compared to the grower units (69%) and the fattening units (60%). This practice was more common (*p* < 0.05) on medium and large farms compared to small farms in regard to the farrowing and grower units, and more common (*p* < 0.05) on large farms compared to small farms in the fattening units. Disinfection between each batch was done in the farrowing units on 59% of the farms and in the grower units on 49% of the farms, which was more common (*p* < 0.05) than in the fattening units (32%). This practice was also more common (*p* < 0.05) on medium and large farms compared to small farms in regard to the farrowing units and more so (*p* < 0.05) on large farms compared to small farms in regard to the grower units. Regarding disinfection between batches in the fattening units, there was no significant difference between the herd sizes (Table 2). A downtime period of a minimum of 4 days between batches was practiced more frequently (*p* < 0.05) in the farrowing units (80%) compared to in grower (66%) or fattening units (67%). Numerically, a downtime period of more than 4 days was more common on medium and large farms compared to small farms, but this could not be verified statistically for all age categories (Table 2).

**Table 2:** Different management practices related to biosecurity, as reported by the surveyed Swedish pig farms, in the farrowing, grower, and fattening sections divided by farm size (for clarification of herd sizes, see Table 1). Regarding total numbers, different letters (a-c) within a column indicate a statistical difference (*p* < 0.05) between age categories. Regarding herd sizes, different letters (d-e) within a row indicate a statistical difference (*p* < 0.05) between herd sizes. The differences were compared statistically using Chi-square tests. When numbers are written in italics, the distribution of the responses did not allow for chi-square analysis

	Total		Herd size					
			Small		Medium		Large	
	n	%	n	%	n	%	n	%
**Cleaning between each batch**								
Farrowing units	106/132	**80**^**a**^	13/24	**54**^**d**^	69/80	**86**^**e**^	24/28	**86**^**e**^
Grower units	91/132	**69**^**b**^	11/24	**46**^**d**^	55/80	**69**^**e**^	25/28	**89**^**f**^
Fattener units	71/119	**60**^**b**^	14/33	**42**^**d**^	*45/74*	***61***	12/12	**100**^**e**^
**Disinfection between each batch**								
Farrowing units	78/132	**59**^**b**^	9/24	**38**^**d**^	50/80	**63**^**e**^	19/28	**68**^**e**^
Grower units	65/132	**49**^**b**^	7/24	**29**^**d**^	40/80	**50**	18/28	**64**^**e**^
Fattening units	38/119	**32**^**a**^	9/33	**27**	27/74	**36**	*2/12*	***17***
**Downtime ≥ 4 days between each batch**								
Farrowing units	106/132	**80**^**a**^	15/24	**63**^**d**^	67/80	**84**^**e**^	*24/28*	***86***
Grower units	87/132	**66**^**b**^	12/24	**50**^**d**^	53/80	**66**	22/28	**79**^**e**^
Fattening units	80/119	**67**^**b**^	18/33	**55**	52/74	**70**	*10/12*	***83***

In the dry sow sections, cleaning between each batch was carried out on 9% (9/104) of farms with deep litter straw beds and on 11% (5/45) of farms with conventional pens (Table 3), which was less common (*p* < 0.05) than commonly practiced in farrowing, grower and fattening units. Disinfection between each batch was carried out on 11% (11/104) of farms with deep litter straw beds and 20% (9/45) of farms with conventional pens. Again, this was significantly (*p* < 0.05) less practiced compared to what was done in the farrowing, grower and fattening units. Numerically it was more common to clean and disinfect between batches of dry sows, indifferent of pen type, on small farms compared to medium and large sized farms (Table 3). A greater proportion of medium and large farms disinfected the pens rather than cleaned them between each batch. It was less common with a downtime of a minimum of 4 days in the dry sow units, indifferent of pen types, compared to the farrowing, grower and fattening units (Table 3) (*p* < 0.05).

**Table 3:** Different management practices related to biosecurity, as reported by the surveyed Swedish pig farms, in the dry sow sections (deep litter straw or conventional pens with limited bedding material) divided by farm size (for clarification of herd sizes, see Table 1). Different letters within a row indicate a statistical difference (*p* < 0.05) between herd sizes. The differences were compared statistically using Chi-square tests. When numbers are written in italics, the distribution of the responses did not allow for chi square analysis

	Total		Herd size					
			Small		Medium		Large	
	n	%	n	%	n	%	n	%
**Cleaning between each batch**								
Deep litter straw	9/104	**9**	*6/14*	***43***	*3/66*	***5***	*0/24*	***0***
Conventional pens	5/45	**11**	*3/7*	***43***	*2/22*	***9***	*0/16*	***0***
**Disinfection between each batch**								
Deep litter straw	11/104	**11**	*3/14*	***21***	*5/66*	***8***	*3/24*	***13***
Conventional pens	9/45	**20**	*2/7*	***29***	*3/22*	***14***	*4/16*	***25***
**Downtime ≥ 4 days between each batch**								
Deep litter straw	43/104	**41**	3/14	**21**^**b**^	25/66	**38**^**b**^	15/24	**63**^**a**^
Conventional pens	12/45	**27**	*1/7*	***14***	*6/22*	***27***	*5/16*	***31***

### Parasite control

Routine faecal testing to determine parasite infections in sows was overall done on 4% of the farms. The corresponding figures for suckling piglets, growers, fatteners, and replacement stock were 3, 4, 2 and 4%, respectively.

**Fig. 2:**
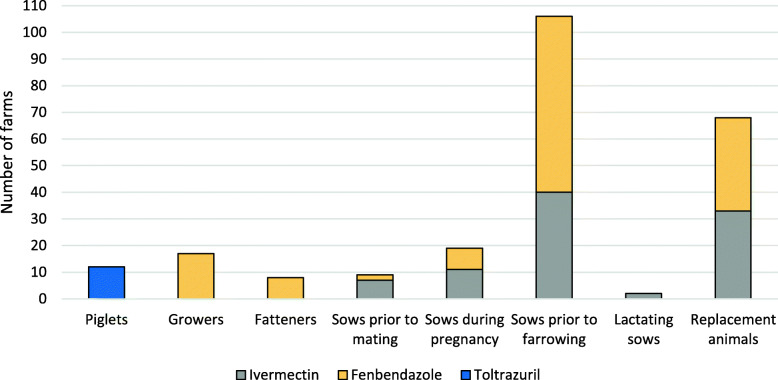
The reported use of antiparasitic drugs shown by age category and stage of production as reported by the surveyed Swedish pig farms. A total of 120/174 farms reported to use antiparasitic drugs that could be administered to several age categories and at several stages of production on the same farm

Overall, antiparasitic drugs were used on 120/174 (69%) of the farms. Out of the 54 farms that did not use antiparasitic drugs, 69% were fattening farms, 12% organic farms, and 19% conventional farrow-to-finish or piglet producing farms. The most common practice was to treat sows prior to farrowing (*n* = 106, corresponding to 79% of all treatments) with either fenbendazole (62%), administered in the feed or with a drench, or with ivermectin (38%) administered as a subcutaneous injection or in the feed. Occasionally anthelmintics were also given to sows at other time-points and 13% of the sow holdings reported on deworming the sows at multiple time points (Fig. 2).

Replacement animals were treated with anthelmintics in 50% (68/135) of the sow holdings and evenly distributed between fenbendazole (24%) and ivermectin (26%). Growers and fatteners were treated with fenbendazole via the feed in 13 and 3% of the farms, respectively. The anticoccidial drug toltrazuril was only used for piglets. It was used in 9% (12/135) of the sow holdings and was administered either directly in the mouth or via the feed (Fig. 2).

Regarding treatments with ivermectin specifically against sarcoptic mange, 68% of the producers stated they had never treated and 11% not for the last 1 to 10 years, whereas 21% treated twice or more per year.

### Health parameters

**Fig. 3:**
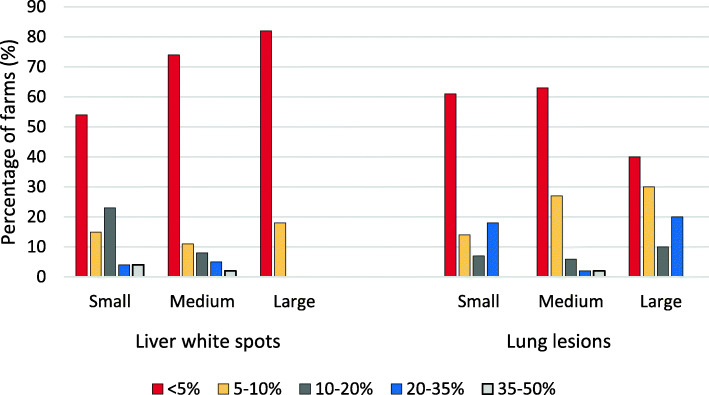
The average percentage of liver condemnations due to white spot lesions, as well as lung condemnations due to pneumonic lung lesions, registered at slaughter over the past 1 year in the surveyed Swedish herds that produced fatteners (liver white spot lesions *n* = 99, lung lesions *n* = 101). The farms were divided based on herd size (for clarification of herd sizes, see Fig. 1)

In total, 119 herds sent pigs to slaughter, 79 farrow-to-finish herds, one central unit in a sow pool and 39 specialised fattening herds. Out of these, 99 farms reported the average percentage of liver condemnations due to white spots, induced by larval migration of *A. suum*, registered at slaughter over the past 1 year. Overall, a majority of farms (74%) reported to have less than 5% of the livers condemned due to white spots (Fig. 3).

**Fig. 4:**
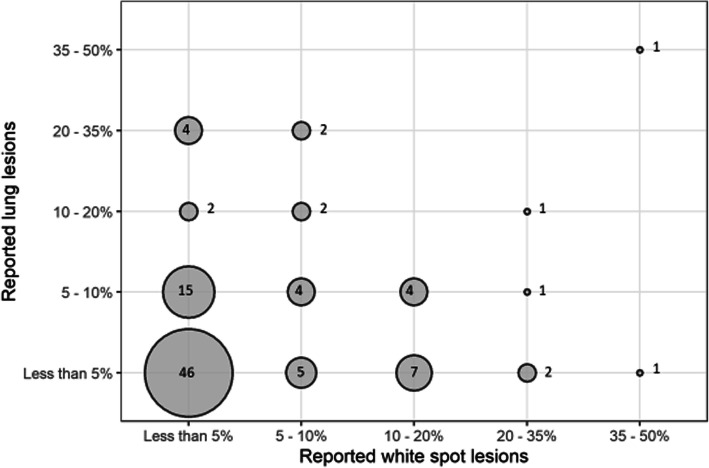
The proportion of reported white spot liver lesions and pneumonic lung lesions, as registered at slaughter, in herds producing fatteners (*n* = 97). The number of herds within each category is shown in the circle

The average percentage of lung condemnations due to pneumonia, registered at slaughter over the past year, was reported by 101 farms. Overall, a majority of farms (65%) reported to have less than 5% of the lungs condemned due to pneumonic lesions (Fig. 3). Ninety-seven farms reported on both liver and lung condemnations at slaughter and the largest proportion of farms (47%, 46/97) had less than 5% of both liver and lung condemnations at slaughter. In contrast, one medium sized herd had more than 35% of both these registrations (Fig. 4).

**Fig. 5:**
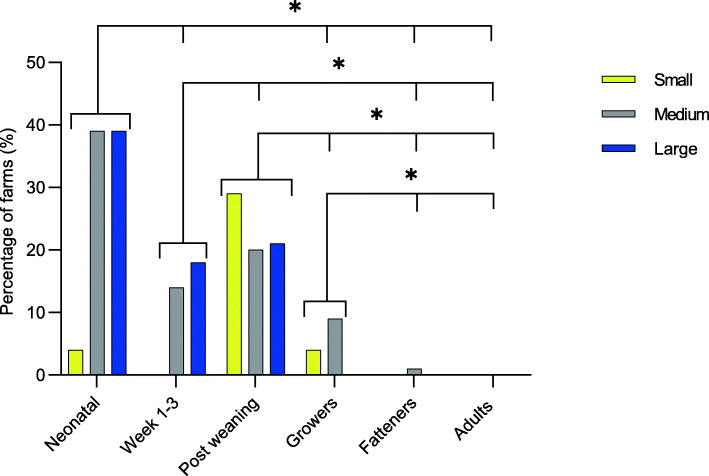
The percentage of farms where diarrhoea was described to occur in all, or in most batches, as reported by the surveyed Swedish farms related to herd size. An asterisk (*) indicates that there is a significant difference (*p* < 0.05) between the age categories when the incidences were compared using chi-square tests. The farms were classified as small if they had fewer than 100 sows or produced less than 5000 fatteners annually (*n* = fatteners 33, other age categories 24), medium if 100–400 sows or produced 5000–10,000 fatteners annually (*n* = fatteners 74, other age categories 80) or large if they had more than 400 sows or produced > 10,000 fatteners annually (*n* = fatteners 12, adults 31, other age categories 28)

Diarrhoea was reported to occur in all or in most batches, in the neonatal period on 33% of the farms. During the sucking period diarrhoea was reported to occur always or in most batches on 13% of the farms and post weaning the figure was 22%. For the growers and fatteners, 6 and 1% of the farms respectively, observed diarrhoea in most baches. In contrast, no farm reported on diarrhoea occurring always or often in adult animals (Fig. 5).

## Discussion

This study documented measures related to the control of gastrointestinal parasites on Swedish pig farms. All data were reported by the producers and are hence based on their perception and their routines. Although the response rate was only 21%, answers were comparably uniform and without many discrepancies, and all types of production types and farm sizes were represented. We therefore concluded that they likely reflected measures undertaken to control gastrointestinal parasites on Swedish pig farms of today. It was shown that 69% of the herds, mainly sow holdings, used antiparasitic drugs routinely. No use was reported in 31% of the herds, mainly specialised fattening herds, but also in organic farms where routine use of antiparasitic drugs is not allowed [27]. Less than 5% of the herds conducted faecal analysis for parasites, and the common use of antiparasitic drugs must hence be regarded as a prophylactic measure undertaken by routine.

The pig roundworm *A. suum* appeared to have limited impact on health and productivity. Most farms had less than 5% white spot liver lesions registered at slaughter, and none of the large farms had more than 10%. Using the degree of white spots as an indicator of *A. suum* infections does however have limitations. In the early infection phase, the degree of white spots appear to correlate well with the level of infection [28], yet, as immunity builds up, larvae are prevented from completing the hepato-tracheal migration route and little or no hepatic scarring will occur [29, 30]. Further, assessing livers is a subjective diagnostic method based on rapid visual inspection at abattoirs, making it an insensitive method for diagnosing *A. suum* infections [31].

Despite that, white spots may be of interest since migrating *A. suum* larvae also can cause lung lesions [32, 33]. Indeed, a positive correlation between registration for white spots and pneumonia at slaughter has been recorded at an individual level [34, 35]. These unspecific measures can therefore be used as a possible indicator of parasite infections at herd level [32]. In this study, only one farm had very high occurrence of both white spot and pneumonia registrations at slaughter, possibly indicating *A. suum* associated pneumonia. However, as most farms reported to have less than 5% of both white spots and lung lesions registered at slaughter, our conclusion was that *A. suum* related problems were limited in the responding herds.

Diarrhoea may occur due to parasitic infections, particularly in piglets infected by *C. suis* or growing pigs infected by *T. suis* [13, 36, 37]. There are however other causes associated with diarrhoea in pigs than parasitic infections [38]. Consequently, without further aetiological investigations, we cannot draw any direct conclusions from the reported degree of diarrhoea on the farms, and to what extent this is related to parasite infections. Nevertheless, the overall incidence of diarrhoea was low, likely indicating a general positive effect of batch wise rearing and the undertaken hygiene measures.

In addition to the use of antiparasitic drugs, suitable management practices have been concluded essential for effective parasite control on pig farms [18]. We investigated several husbandry and biosecurity factors that may affect the survival and transmission of the external life cycle stages of parasites and related them to age categories and herd sizes. Strategic hygiene and biosecurity measures are generally recommended to minimise the number of free-living stages of gastrointestinal parasites in the environment. Nematode eggs, such as those of *A. suum* and *T. suis,* are exceedingly robust and may survive for years in the environment [11]. However, both embryonation and larval development are dependent on external factors such as temperature, humidity, oxygen and pH [11, 17, 39, 40]. Similarly, oocysts from *C. suis* have a high survival capacity and may sporulate when the temperature is 20 °C or above, provided the humidity is adequate [41, 42]. By contrast, the eggs and the early external larval stages of *Oesophagostomum* spp. are more sensitive to environmental factors, whereas the infective third stage (L3) of *Oesophagostomum* spp. is more robust and may survive in the environment for up to a year [11, 18]. From our results, medium, and large sized herds had more strategic hygiene and biosecurity practices compared to small farms. Indeed, it has previously been shown that larger farms are likely to have more intensified production, which also was associated with a reduced occurrence of gastrointestinal parasites, possibly due to the implementing of effective hygienic measures [10, 18].

Age segregated rearing from birth to slaughter has increased in Sweden following the ban of growth promotors in 1986, and has earlier been concluded common in conventional pig herds [3, 43]. In this study, age segregated batch production was commonly practiced, more so in the farrowing and grower sections compared to the fattener sections. Batch production, where young pigs are not mixed with older pigs, has previously been shown to reduce gastrointestinal parasites infections [10, 15, 18, 44]. The frequently practiced batch production may partly explain the findings above regarding a low clinical impact of parasites in the surveyed herds. The low incidence of treatments with toltrazuril provides further support to this conclusion. Toltrazuril is frequently used in the European Union for piglets aged 3–5 days as prophylactic treatment against *C. suis* [25]. In a previous Swedish study, anticoccidial treatment was used in 40% of the investigated herds [45]. In this study however, only 9% of the sow holdings treated piglets with toltrazuril. According to the Summary of Product Characteristics, a confirmed history of *C. suis* infection is required prior to treatment with this drug [46]. The low usage recorded in our study may therefore indicate a limited clinical impact of this parasite in the surveyed herds, despite *C. suis* being relatively prevalent in Swedish pig herds [47].

Adequate cleaning, disinfection, and enough time between batches for the pen to thoroughly dry are all important management practices that reduce parasite survival and transmission [18, 41, 44, 48]. From our observations it appeared that a great proportion of the medium and large farms applied appropriate hygiene measures on the empty farrowing pens between batches, although this was less practiced on small farms. It was common for the farms to have a downtime of at least 4 days between batches in the farrowing sections, which may be protective against the build-up of infective *C. suis* oocysts [41]. Overall, cleaning between batches in the grower and fattening sections was common on all farms. Although the downtime period varied, it was generally more than 4 days. This indicated that the application of adequate hygiene measures between batches of growing pigs was common in the surveyed farms which also was in line with previous reports of good internal biosecurity on Swedish pig farms [49].

For dry sows, deep litter straw pens were the most common pen types. Deep litter straw provides a microenvironment with an unfavourable pH and low oxygen levels unsuitable for parasite development [39, 40]. However, deep litter straw pens generally house more than one farrowing group, resulting in a continuous production system for dry sows. Subsequently this limits the ability to perform regular cleaning between batches, which was clear from our results. Oddly, a greater proportion of the large farms reported on disinfecting between batches of dry sows compared to cleaning between batches. A possible explanation might be that they only disinfected parts of the pens or used dry disinfecting agents without prior thorough cleaning, something that is required to achieve a good effect from the disinfectant.

The water source was placed over the slatted part of the floor for growers and fatteners in the majority of the medium and large sized farms, This may reduce the risk of spilled water creating a damp environment, suitable for parasite survival and embryonation [17]. In small farms it was less common to place the water source over the slats, increasing the risk of wet floor areas. Liquid feed may also increase moisture in the pens. It was used for fatteners by almost all medium and large sized farms and a large proportion also used it for the growers, indicating a possible risk of increased moisture in the pens.

In Sweden, group housing is practiced for pigs of all ages, bedding material is a legal requirement and fully slatted floors are not allowed [5]. These are all housing factors that may facilitate parasite survival and transmission [15, 18, 50–53]. Still, the impact of parasites appeared to be low, most likely due to the common use of age segregated rearing of growing pigs and the strategic hygiene measures undertaken between.

Very few of the participating farms provided outdoor access and the ones that did were either small herds or farms with organic production. It has been reported that access to the outdoors may favour parasite survival and transmission [11, 26, 54].

Correct use of antiparasitic drugs is important to ensure good parasite control. Underdosing or treating pigs at un-strategic timepoints may not only result in treatment failure but may also select for resistance [20, 23]. The most common practice in the surveyed farms was to treat sows prior to farrowing with either fenbendazole or ivermectin. Treating sows prior to farrowing could be defined to be strategic as it reduces possible contamination of the farrowing pen, especially when combined with pre-cleaning as was carried out in most herds. Altogether, this illustrated a generalised focus within the surveyed farms on preventing parasite transmission to the piglets, which also was elucidated by the common use of anthelmintics in sow holdings and the rare use in specialised fattening farms.

Still, frequent use of anthelmintics without prior knowledge of the parasite status in the herds represents a risk factor for selection of resistance [55], especially considering that fenbendazole and ivermectin are the only anthelmintic substances available for use in pigs in Sweden. If management routines are likely to solely control parasites in a herd, regular prophylactic anthelmintic treatment can be discontinued. Instead, routine faecal monitoring for parasites could be done, and treatment only instituted when deemed necessary [9]. This would also allow for monitoring of anthelmintic efficiency. Since gastrointestinal parasites mainly cause subclinical infections, combined with the convenience of regular use of antiparasitic drugs, many farmers may not be motivated to change management and hygiene practices in order to reduce the parasite occurrence on their farm [11]. It must however be considered that inappropriate hygiene measures, resulting in survival of parasite eggs and larvae in the environment, will result in continual re-infections despite the regular use of antiparasitic drugs [17].

## Conclusion

In this study we assessed management practices related to the control of gastrointestinal parasites on Swedish pig farms. Several risk factors for parasite infections, such as bedding material, group housing and solid floors, are mandatory requirements by national law. However, it was evident from this study that although strategic hygiene and biosecurity practices appeared common, they were not practiced in all herds and less so for growing pigs on small farms for dry sows in herds of all sizes. The use of antiparasitic drugs was frequent and mainly carried out through routine prophylactic treatments of sows prior to farrowing. Regular faecal testing for parasites was however uncommon. A holistic approach is desirable when designing efficient parasite control programs, and it is essential that management factors and routine monitoring of parasites are given attention. This not only to achieve good parasite control on the farm, but also to reduce the risk of unnecessary use of antiparasitic drugs.

## Supplementary Information

**Additional file 1.** English translation of the web-based questionnaire “Avmaskningsrutiner i svenska grisbesättningar”.

## Data Availability

The datasets analysed during the current study are available from the corresponding author on reasonable request.
