# Supplementary material for: Management practices related to the control of gastrointestinal parasites on Swedish pig farms
Source: Porcine Health Manag. 2021 Jan 20;7:12. doi: 10.1186/s40813-021-00193-3 (PMC7816406; doi:10.1186/s40813-021-00193-3)
Supplement: Supplementary file 1 — Additional file 1. English translation of the web-based questionnaire “Avmaskningsrutiner i svenska grisbesättningar”. [file 40813_2021_193_MOESM1_ESM.pdf]

English translation of the web-based questionnaire “Avmaskningsrutiner i svenska grisbesättningar”.

Parasite control routines in Swedish pig herds

The aim with this study is to document the herd structures and management routines that can be related to parasite control on Swedish pig farms. The parasite status of Swedish pigs has not been investigated since the 1980s, and a lot has changed in Swedish pig production since then. Today we know very little of the actual parasite status of Swedish pigs and there is also hardly any information on how herds control and manage parasites. The goal with this questionnaire, and a subsequent prevalence study, is to gain information and knowledge that can form a base for new and updated recommendations on parasite control, suitable for modern pig production.

It takes approximately 20 minutes to fill out this questionnaire.

1) What type of herd do you have?

- ○ Farrow-to-finish
- ○ Specialised piglet producer
- ○ Specialised fattener producer
- ○ Central unit in a sow pool
- ○ Satellite in a sow pool, farrow-to-finish
- ○ Satellite in a sow pool, specialised piglet producer

2) What type of production do you have?

- ○ Conventional
- ○ KRAV-certified or organic
- ○ EU-organic
- ○ Specific Pathogen Free (SPF)
- ○ Outdoor
- ○ Other

3) What herd size do you have?

- ○ No sows, the herd is a specialised fattening herd
- ○ Less than 100 sows, or a satellite in a sow pool with less than 220 farrowings/year
- ○ 100 - 400 sows, or a satellite with 220-880 farrowings/year
- ○ More than 400 sows, or a satellite with more than 880 farrowings/year

4) How many fatteners are produced each year?

- ○ 0 (I do not have fatteners)
- ○ Less than 1500
- ○ 1 500 - 5 000
- ○ 5 000 - 10 000
- ○ 10 000 - 20 000
- ○ More than 20 000

5) What type of pens do you have?

- ☐ Conventional farrowing pens
- ☐ Conventional grower pens
- ☐ Unit pens (weaned piglets remain in the farrowing pens)
- ☐ Multi-litter pens for growers
- ☐ Family-pens (several sows with their piglets)
- ☐ Farrow-to-finish pens
- ☐ Conventional fattening pens
- ☐ Deep litter straw pens (fatteners)
- ☐ Unspecified fattening pens
- ☐ Deep litter straw pens (dry sows)
- ☐ Conventional dry sow pens
- ☐ Other

6) Do you practice age segregated, batch wise production?

	Always	Mostly	No	n/a
For piglets	○	○	○	○
For growers	○	○	○	○
For fatteners	○	○	○	○

7) What type of bedding material is used? More than one option can be selected.

	n/a	Straw	Peat	Wood shavings	Deep litter	Other
For piglets	☐	☐	☐	☐	☐	☐
For growers	☐	☐	☐	☐	☐	☐
For fatteners	☐	☐	☐	☐	☐	☐
For dry sows	☐	☐	☐	☐	☐	☐

8) Do you use dry or wet feed?

	n/a	Dry	Wet	Both
For growers	☐	☐	☐	☐
For fatteners	☐	☐	☐	☐
For dry sows	☐	☐	☐	☐

9) How is the water supplied?

	n/a	Automatic waterers	Nipple drinkers	Both	Other
For piglets	○	○	○	○	○
For growers	●	○	○	○	○
For fatteners	●	○	○	○	○
For dry sows	●	○	○	○	○

10) Where is the water facility placed? More than one option can be selected.

	n/a	Over the slats	Over the solid floor	Over feed trough
For piglets	☐	☐	☐	☐
For growers	☐	☐	☐	☐
For fatteners	☐	☐	☐	☐
For dry sows	☐	☐	☐	☐

11) At what age are the piglets weaned?

- n/a
- 4–5 weeks
- 5–6 weeks
- 6–7 weeks
- Older than 7 weeks

12) For how long do the piglets stay in the farrowing pens?

- We do not have piglets
- We have unit pens
- Moved directly at weaning
- Moved 1 week after weaning
- Moved 2 weeks after weaning
- Moved 3 weeks after weaning

13) At what age are the growers moved to the fattening units, or sold?

- ○ n/a
- ○ 8 weeks or younger
- ○ 9 weeks
- ○ 10 weeks
- ○ 11 weeks
- ○ 12 weeks
- ○ 13 weeks
- ○ 14 weeks or older

14) What is the estimated weight when the growers are moved to the fattening units, or are sold?

- ○ n/a
- ○ Less than 23kg
- ○ 23-26kg
- ○ 27-29kg
- ○ 30-32kg
- ○ 33-35kg
- ○ More than 35kg

15) After how many weeks in the fattening units are the first animals sent to slaughter?

- ○ n/a
- ○ 10 weeks or earlier
- ○ 11 weeks
- ○ 12 weeks
- ○ 13 weeks
- ○ 14 weeks
- ○ 15 weeks
- ○ More than 15 weeks

16) After how many weeks in the fattening units are the last animals sent to slaughter?

- n/a
- 13 weeks or earlier
- 14 weeks
- 15 weeks
- 16 weeks
- 17 weeks
- 18 weeks
- More than 18 weeks

17) What is the estimated slaughter weight?

- n/a
- 75kg or less (100kg or less live weight)
- 75-79kg (100-105kg live weight)
- 80-82kg (106-110kg live weight)
- 83-86kg (111-115kg live weight)
- 87-90kg (116-120kg live weight)
- 91-93kg (121-125kg live weight)
- 94-96kg (126-130kg live weight)
- More than 96kg (more than130kg live weight)
- Do not know

18) Do any pigs have outdoor access?

	n/a	Yes	No
Nursing piglets	○	○	○
Growers	○	○	○
Fatteners	○	○	○
Dry sows	○	○	○

19) How often are the following pens washed?

	n/a	Before every new batch	Before every second batch	Before every third batch	Less frequently	Never
Farrowing pens	○	○	●	○	○	○
Grower pens	○	○	●	○	○	○
Family pens	○	○	●	○	○	○
Farrow-to-finish pens	○	○	○	○	○	○
Fattening pens	○	○	○	○	○	○
Deep litter straw beds	○	○	○	○	○	○
Conventional dry sow pens	○	○	●	○	○	○

20) How often are the following pens disinfected?

	n/a	Before every new batch	Before every second batch	Before every third batch	Less frequently	Never
Farrowing pens	○	○	○	○	○	○
Grower pens	○	○	○	○	○	○
Family pens	○	○	○	○	○	○
Farrow-to-finish pens	○	○	○	○	○	○
Fattening pens	○	○	○	○	○	○
Deep litter straw beds	○	○	○	○	○	○
Conventional dry sow pens	○	○	○	○	○	○

21) What disinfectant(s) do you use? If you do not disinfect you can leave this space blank.

22) How long downtime do the following pens have?

	n/a	0	1–3 days	4-6 days	7-10 days	More than 10d	Varies
Farrowing pens	○	○	○	○	○	○	○
Grower pens	○	○	○	○	○	○	○
Family pens	○	○	○	○	○	○	○
Farrow-to-finish pens	○	○	○	○	○	○	○
Fattening pens	○	○	○	○	○	○	○
Deep litter straw beds	○	○	○	○	○	○	○
Conventional dry sow pens	○	○	○	○	○	○	○

The antiparasitic drugs available in Sweden are:

A) Avermectins such as Ivomec, Noromectin and Bimectin

B) Benzimidazoles such as Axilur, Panacur and Zerofen

C) Triazine compounds such as Baycox and Zorabel

23) Are any of the sows treated with any antiparasitic drugs? If yes, what drug is used, and how is it administered?

	Drug class (see above)						Administration route				
	n/a	No treatment	A	B	C	Other	n/a	In feed	In water	Drench	Injection
Before insemination											
During pregnancy											
Before farrowing											
During suckling											

24) Are pigs of any other ages treated with antiparasitic drugs? If yes, what drug is used, and how is it administered?

	Drug class (see above)					Administration route				
	n/a	No treatment	A	B	C	n/a	In feed	In water	Drench	Injection
Nursing piglets										
Growers										
Fatteners										
Replacement animals older than 6 months										

25) is faecal analysis carried out to check for parasites?

	n/a	Never	Sometimes	Often
Sows	◯	◯	◯	◯
Nursing piglets	◯	◯	◯	◯
Growers	◯	◯	◯	◯
Fatteners	◯	◯	◯	◯
Replacement animals older than 6 months	◯	◯	◯	◯

26) If sarcoptic mange has not been eradicated in the herd, how often to treat for this?

- ◯ Never
- ◯ Twice or more per year
- ◯ 1 time per year
- ◯ More than 1–3 years ago
- ◯ More than 3–5 years ago
- ◯ More than 5 years ago

27) How often does diarrhoea occur in the following pigs?

	n/a	Always	Most batches	Occasional batches	Never	Do not know
Piglets during the first week	☐	☐	☐	☐	☐	☐
1-3 week old piglets	☐	☐	☐	☐	☐	☐
Piglets at weaning	☐	☐	☐	☐	☐	☐
Growers	☐	☐	☐	☐	☐	☐
Fatteners	☐	☐	☐	☐	☐	☐
Adults	☐	☐	☐	☐	☐	☐

28) Is zinc oxide used in the feed?

- ◯ Never

- Not the past 12 months
- Sometimes
- Always at weaning

The larvae of the pig roundworm migrate through the body of the pig and may then cause damage to the liver and the lungs. This damage may be noted at slaughter.

29) What proportion (mean value over the past year) of livers are condemned at slaughter due to parasitic liver damage/white spots (code 83/84)?

- Do not have fatteners
- Less than 5%
- 5 - 10%
- 10 - 20%
- 20 - 35%
- 35 - 50%
- More than 50%
- Do not know

30) What proportion (mean value over the past year) of pneumonic lesions are registered at slaughter (code 61/62)?

- Do not have fatteners
- Less than 5%
- 5 - 10%
- 10 - 20%
- 20 - 35%
- 35 - 50%
- More than 50%
- Do not know

Information and a question regarding part two of this project

In a second part of this project, we will be collecting faecal samples from pigs of different age categories to look for gastrointestinal parasites

31) Would you be interested in participating in part two of this study and send in faecal samples?

- ○ Yes
- ○ No

All of you who have answered yes on question 31 and are interested in participating in part two of this study, please fill in your contact details (name, address, phone number and email address) so that we are able to contact you.

If you are selected to be in the second part of the study, sampling material will be sent out to you together with detailed information about sampling. The samples will be analysed at the National Veterinary Institute and you will of course be notified of the results from your herd. For you this means up to 50 free faecal samples, In the final report the results will however be anonymous.

Results will be disseminated through Grisföretagaren.

32) Contact details:

© Copyright www.questback.com. All Rights Reserved.
